# Recent experience impacts social behavior in a novel context by adult zebrafish (*Danio rerio*)

**DOI:** 10.1371/journal.pone.0204994

**Published:** 2018-10-18

**Authors:** Delawrence J. Sykes, Piyumika S. Suriyampola, Emília P. Martins

**Affiliations:** 1 Department of Biology, Indiana University, Bloomington, Indiana, United States of America; 2 Molecular and Integrative Physiology, University of Michigan, Ann Arbor, Michigan, United States of America; 3 School of Life Sciences, Arizona State University, Tempe, Arizona, United States of America; University of Portsmouth, UNITED KINGDOM

## Abstract

Many animals exhibit behavioral plasticity as they move between habitats seasonally, reside in fluctuating environments, or respond to human-induced environmental change. We know that physical environment during early development can have a lasting impact on behavior, and on the neural mechanisms that shape behavior. In adults, social context can have similar persistent effects on behavior and the brain. Here, we asked whether physical context impacts adult social behavior in a novel environment. We placed groups of adult zebrafish (*Danio rerio*) in two different physical contexts. After two weeks, we measured group behavior in a novel context, and found that zebrafish with recent experience in a more-complex physical environment charged each other more often and tended to form tighter shoals than did fish that had been housed in less-complex environments. These differences were present regardless of the novel context in which we assayed behavior, and were not easily explained by differences in activity level. Our results demonstrate the impact of recent experiences on adult behavior, and highlight the importance of physical as well as social history in predicting animal behavior in novel situations.

## Introduction

Many adult animals move in and out of various physical contexts seasonally or in response to disturbance, a feature that has become increasingly important with human-induced shifts in habitat and climate [1, 2, 3]. Experiences with different physical contexts during early development can have dramatic and prolonged effects on behavior, shaping underlying neural and other systems in ways that influence later adult behavior [4, 5]. However, adult behavior is also plastic and can be influenced by a variety of factors including physical context [6, 7, 8]. Here, we use experiments with zebrafish to ask whether two weeks of exposure to particular physical contexts influences how animals respond in a novel situation.

As we have long known from studies of critical periods, experiences during early development can have pronounced effects on adult behavior [4, 9]. Recent studies have emphasized the importance of early stress and maternal effects mediated by hormonal and epigenetic mechanisms. For example, treating zebra finches with stress hormones early in development can have profound impacts on later social preferences [10]. Similarly, the amount of parental care received by young animals can impact later stress response [11] and levels of aggression [12]. More general aspects of the physical context can also be important. For example, young trout developing in low density conditions showed higher adult survival than those in high densities [13]. Here, we ask about the importance of experiences during adulthood.

We know that some types of adult experiences can also have both immediate and long-term effects on adult behavior [14, 15]. For example, adult zebrafish housed in mixed strain groups were more aggressive than were those housed in pure-strain groups even one month after the experience [16]. Similarly, adult male guppies spending five weeks in a male-biased social environment showed less mating effort and were more likely to engage in courtship rather than sneak copulations than were those spending the same amount of time in female-biased contexts [17]. Social interactions can have important impacts on brain physiology that may impact future behavior. For example, female cichlids that observed their male partners win a battle showed increased gene expression in regions of the brain associated with reproduction, whereas those who observed their male partners lose a battle showed increased gene expression in regions associated with anxiety [18]. It is not clear, however, whether physical contexts can have similarly persistent effects on adult behavior.

Clearly, the physical environment can have an immediate impact on adult behavior. For example, increasing structural complexity can decrease [19–21] or increase aggression [22]. Drastic contrast between physical environments is not necessary for highlighting this effect; simply adding bricks [23] or gravel [24] to an animal’s surroundings can be sufficient to decrease the frequency of aggressive encounters. Similarly, relatively small increases in turbidity can decrease the size of guppy social groups [25], and increasing the amount of available space can impact near-neighbor distances even when the animals are not crowded [26]. These sorts of findings have had important implications for management and welfare of captive animals and may give insight into predictions of the response of animals to human-induced environmental change [27]. In addition to influencing aggression, environmental enrichment generally decreases the frequency of species atypical behavior and repetitive anxiety-like behavior in a range of animal taxa [28]. Here, we ask whether recent experience in a particular environment has a persistent impact on social behavior.

We use zebrafish, an important model organism for genetic, developmental, and behavioral research [29], as our study organism. For example, emerging research takes advantage of translucent larvae for optogenetics [30] and exploits the regenerative ability of adults to uncover the switch between quiescent and proliferative phenotypes paramount in understanding cardiomyocyte development [31]. Recent behavioral research has identified key genes associated with behavior [32], and has begun exploring sophisticated questions such as the mechanisms underlying numerical abilities [33] and the effects of chronic stress on social behavior [34, 35].

In this study, we ask whether a recent, short-term experience in an enriched physical context can have a persistent impact on adult social behavior. We placed zebrafish in less complex (an empty aquarium) or more complex (with plastic plants and pots) physical contexts for two weeks and then tested their social behavior in two novel contexts differing in complexity. In addition, we measured activity levels to determine whether observed differences in social behavior can be easily explained by changes in overall activity. By testing zebrafish in two assay contexts (less and more complex), we compared the effects of recent experience with the impact of current context in determining social behavior.

## Materials and methods

### Subjects

We used adult zebrafish (> 6 months) of the wild-type, outbred, SH strain bred by Aquatica Biotech (Florida USA). This strain has been used in several previous studies of zebrafish behavior [16, 26, 36, 37]. Before the start of our experiment, we maintained the fish in large groups (20–30 individuals in 38 L aquaria) for about 14 days. We then created groups of 6 fish by combining fish mostly from the same colony tanks to maximize familiarity. During the experiment, we housed these groups of six fish in separate 20.8 L (5.5 gallon) aquaria, and standard conditions: 28° C, 14:10 hour light/dark cycle, and *ad libitum* Tetramin flake food (daily, at roughly the same mid-late morning time whether the fish were in experience treatments or in assay tests). This study was approved by Indiana University’s Institutional Animal Care and Use Committee as part of protocol number 15–017.

### Recent experience in more or less complex environments

To begin the experiment, we placed groups of adult zebrafish into either more- or less- complex physical environments, leaving them to interact with each other and with their environments during 14–17 days. In the wild, zebrafish in still-water populations form groups of 4–22 individuals (average shoal diameters ranging from 11–27 cm) in waters that range from 0 to 50% vegetation cover [38]. Thus, in the current study, we formed groups of 6 zebrafish (3 males and 3 females), placing 16 of these groups into less-complex physical environments, each consisting of a 20.8L aquarium containing only a small submersible filter. In addition, we formed 14 groups and placed them in more-complex physical environments, with half of a 11-cm clay pot and three sections of plastic plants (All Living Things Turtle Grass) in addition to the small submersible filter. Together, the pots and small plants occupied less than 15% of the horizontal area of each aquarium, and the small filters produced weak flows that did not differ across the two treatment conditions. We then allowed each group of fish to interact with each other and with their environments during 14–17 days.

### Behavioral assays

After two weeks of experience in more- or less-complex contexts, we measured the behavior of each group in two novel testing arenas over four consecutive days. Each testing arena was a 20.8 L aquarium, which we made novel by adding a white gravel substrate and submersible filter, and by lowering the water level to 11 cm. The submersible filters again produced a weak flow that was the same as in the two-week experience treatment. The white background and lower water level facilitated automatic video-tracking in two dimensions (see below). Since adult zebrafish prefer dark over light environments [39], this testing context may have been both novel and mildly stressful. We used two testing arenas to compare behavior in novel versions of more and less complex environments. For the less-complex testing context, we assayed fish behavior in the white testing arena containing only the submersible filter and white gravel substrate. For a more-complex novel context, we also tested fish in the same testing arena to which we had added four pieces of curved white plastic (cut PVC tubes) that (like the plastic plants above) provided an element of physical complexity. Because behavior is often highly variable, we measured each group a total of four times: twice in each of the two novel contexts.

After placing each group in its assay aquarium, we waited for a 24-h acclimation period and then video-recorded group behavior from above for 6 min with a Logitech c920 HD Pro Webcam. We then immediately removed or added the cut plastic PVC to create the second assay context, waited a second 24-h acclimation period, and filmed a second 6-min trial. We then repeated the entire process, until we had recorded each group during a total of four 6-min trials. We tested 16 groups (chosen at random) first in the PVC assay followed by the "No PVC" assay, whereas the remaining 14 groups received the two assays in reverse order. Because adult zebrafish tend to be more active in the early morning, we conducted all trials between 8:00 am and 12:00 pm EST.

### Behavior tracking and scoring

We minimized observer bias by using EthoVision XT10 [40] to determine the x and y coordinates of each of the 6 fish in a two-dimensional space every 0.06 s (1000 moments per min). We then scored behavior during these behavioral assays in terms of Group Diameter, *Charge Rate*, and *Activity*. Following Suriyampola et al. [41], we defined charges as the number of episodes of fast movement (> 20 cm/s) of any one fish towards another (distance > 0.3 cm), and then scored *Charge Rate* as the number of charges by any fish in the group during one minute. Way et al. [42] characterized many different kinds of zebrafish behavior that could be considered aggressive including bites, chases, lateral displays, charges, and darts. Our estimates of *Charge Rate* are related to the “charges” described by Way et al. [42], differing in that we counted each episode occurring in 1000 moments / min as a separate charge, such that the total sum of charges scored using EthoVision was substantially larger than what a human observer would have scored, reflecting also the duration of each episode. We also counted only those episodes in which the charging fish traveled more than a minimum distance (0.3 cm).

Charges are a likely measure of aggression, since they are usually produced by more dominant individuals, and the recipient of a charge generally flees. However, zebrafish behavior may also depend on the cohesion of the social group, with fish that are more tightly associated with each other in physical space interacting with each other more often than those that are spread further apart. We thus also estimated *Group Diameter* at each tracked moment by using the rgeos [43] function in R [44] to estimate the maximum distance between any two fish in the group at each point in time. Finally, zebrafish are in near-constant motion, such that increased *Charges* may be a simple consequence of increased activity. To test this possibility, we also estimated *Activity* as the average distance moved (cm) from one moment to the next, summing for all six fish in each group.

### Statistical analyses

For each behavioral measure (*Group Diameter*, *Charge Rate*, and *Activity*), we used two-way ANOVA models to test for the effects of recent experience (more- or less–complex contexts), including also factors indicating novel assay type (PVC or No PVC) and the interaction between experience and assay type. We use repeated-measures rather than standard ANOVA in order to take into account that each group was tested four times (twice in each assay type).

Second, we calculated Pearson product-moment correlation coefficients to assess the similarity between behavioral measures of each group in the same assay type on different days of the experiment. Note that degrees of freedom vary somewhat between analyses because we excluded some trials in which the video quality was inadequate for accurate tracking. We conducted all calculations using the base commands of the R statistical package [44], including residual analyses to confirm the usual ANOVA assumptions of homoscedasticity and normality.

## Results

**Fig 1:**
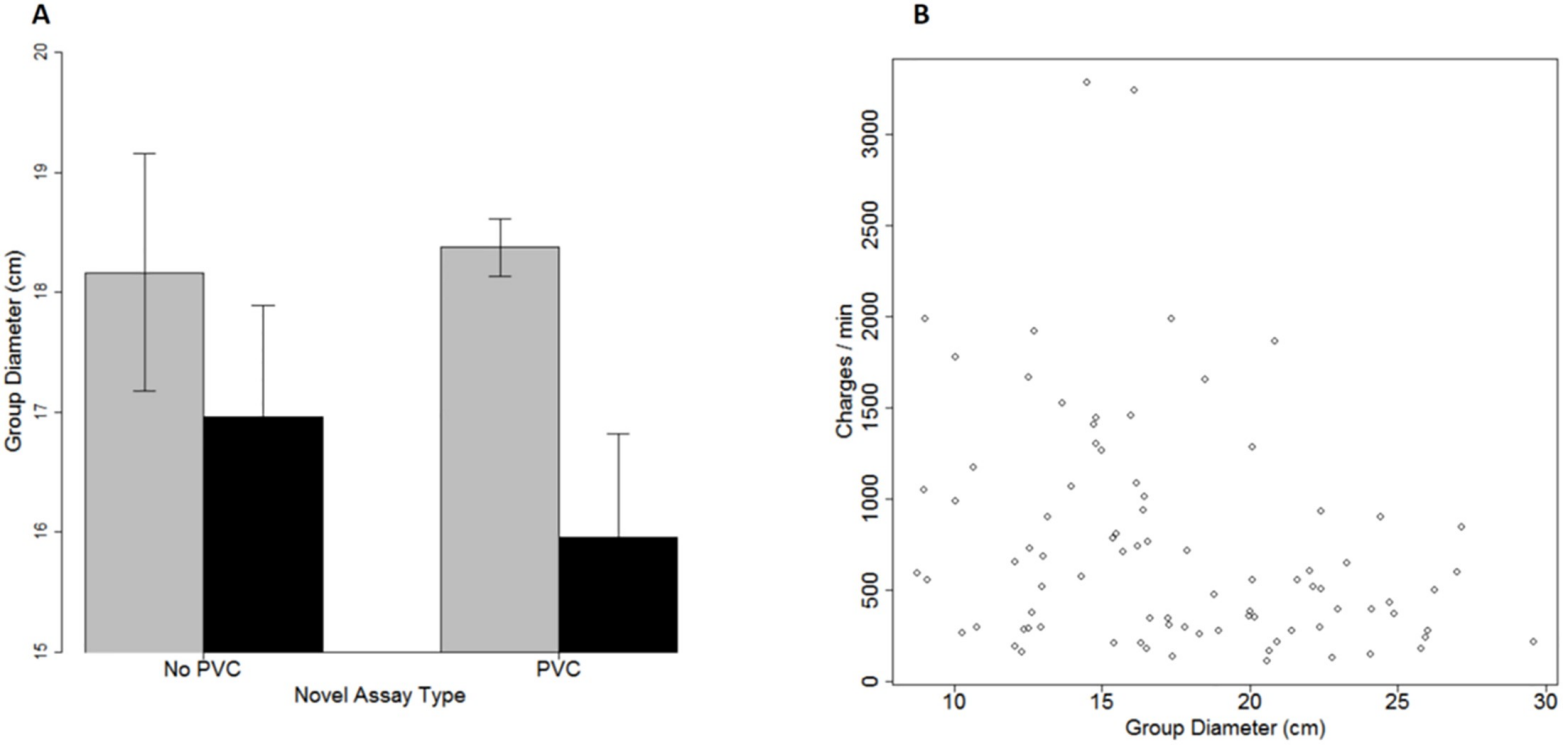
(a) Zebrafish groups with recent experience in a more-complex environment (black bars) also shoaled more closely together than did fish with recent experience in a less-complex environment (gray bars). This pattern was weaker in one novel assay context (No PVC) than in the other, yielding a significant interaction effect (*F*_1,28_ = 4.9, *P* < 0.04). Error bars represent ± 1 standard error. (b) Zebrafish in tighter shoals (with smaller group diameters) charged more frequently, such that there was a moderate negative relationship between *Group Diameter* and *Charge Rate*.

Zebrafish with recent experience in a more-complex environment shoaled more closely together, forming shoals that were 1–2 cm smaller in diameter than did fish with recent experience in a less-complex environment (Fig 1a). This effect was stronger in the more-complex PVC assay (Fig 1a two bars on the right) than in the less-complex, No-PVC assay (Fig 1a two bars on the left), leading to a significant interaction between recent experience and assay type in our two-way, repeated measures ANOVA (*F*_1,28_ = 4.9, *P* = 0.04). Main effects of recent experience (*F*_1,28_ = 1.9, *P* = 0.17), assay type (*F*_1,28_ = 0.1, *P* = 0.79) and within-group effects (*P* > 0.6) were not statistically significant. Zebrafish groups with more cohesive shoals (shorter *Group Diameter*) charged more often, leading to a negative correlation between these two variables (*r* = -0.3, df = 83, *P* = 0.005; Fig 1b). More generally, none of the groups of fish that were widely dispersed charged each other often (Fig 1b), suggesting that proximity may be an important prerequisite to frequent charges.

**Fig 2:**
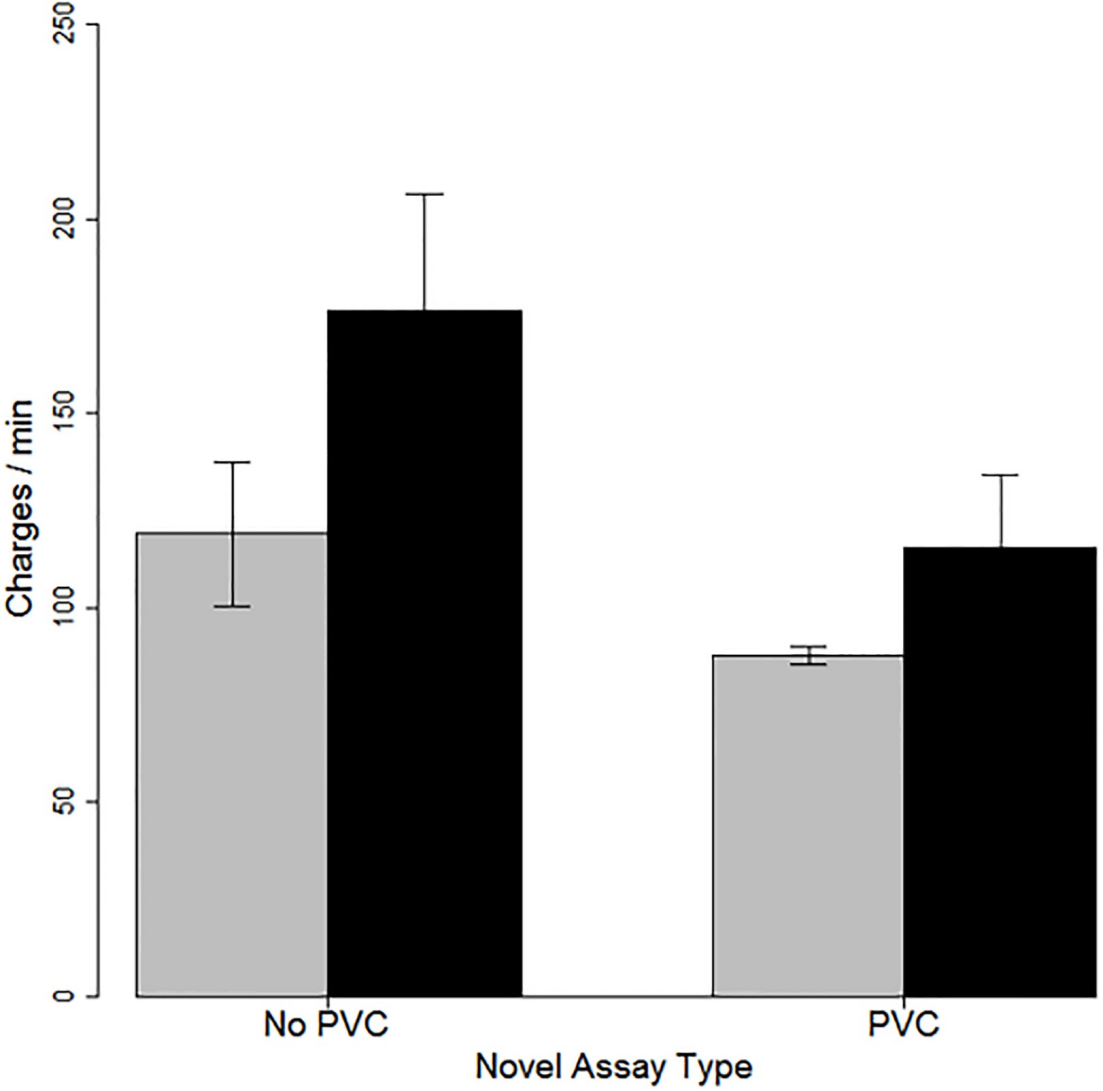
Zebrafish with recent experience in a more-complex environment (black bars) charged each other marginally more often than did fish with recent experience in a less-complex environment (gray bars). Assay type had the opposite effect: fish tested in arenas with complex physical structure (PVC: two bars on the right) charged less often than did fish tested in empty arenas (two bars on the left). Error bars represent ± 1 standard error.

Zebrafish groups with experience in more-complex environments also charged more often (150 ± 21.4 charges / min) than did those with experience in less-complex tanks (103 ± 11.2 charges / min), regardless of the novel assay type (Fig 2). However, *Charge Rates* were quite variable, especially when measured in the empty assay, so the main effect of recent experience on *Charge Rate* was only marginally significant (*F*_1,28_ = 3.5, P = 0.07) in our repeated-measures ANOVA. Assay type also had a marginally-significant impact on *Charge Rate* (Fig 2), albeit in the opposite direction, with zebrafish charging more often when tested in empty arenas than when tested in arenas with cut PVC pipe (within group effect: *F*_1,51_ = 3.5, *P* = 0.07). All other main, interaction and within-group effects were not statistically significant (*P* > 0.3).

**Fig 3:**
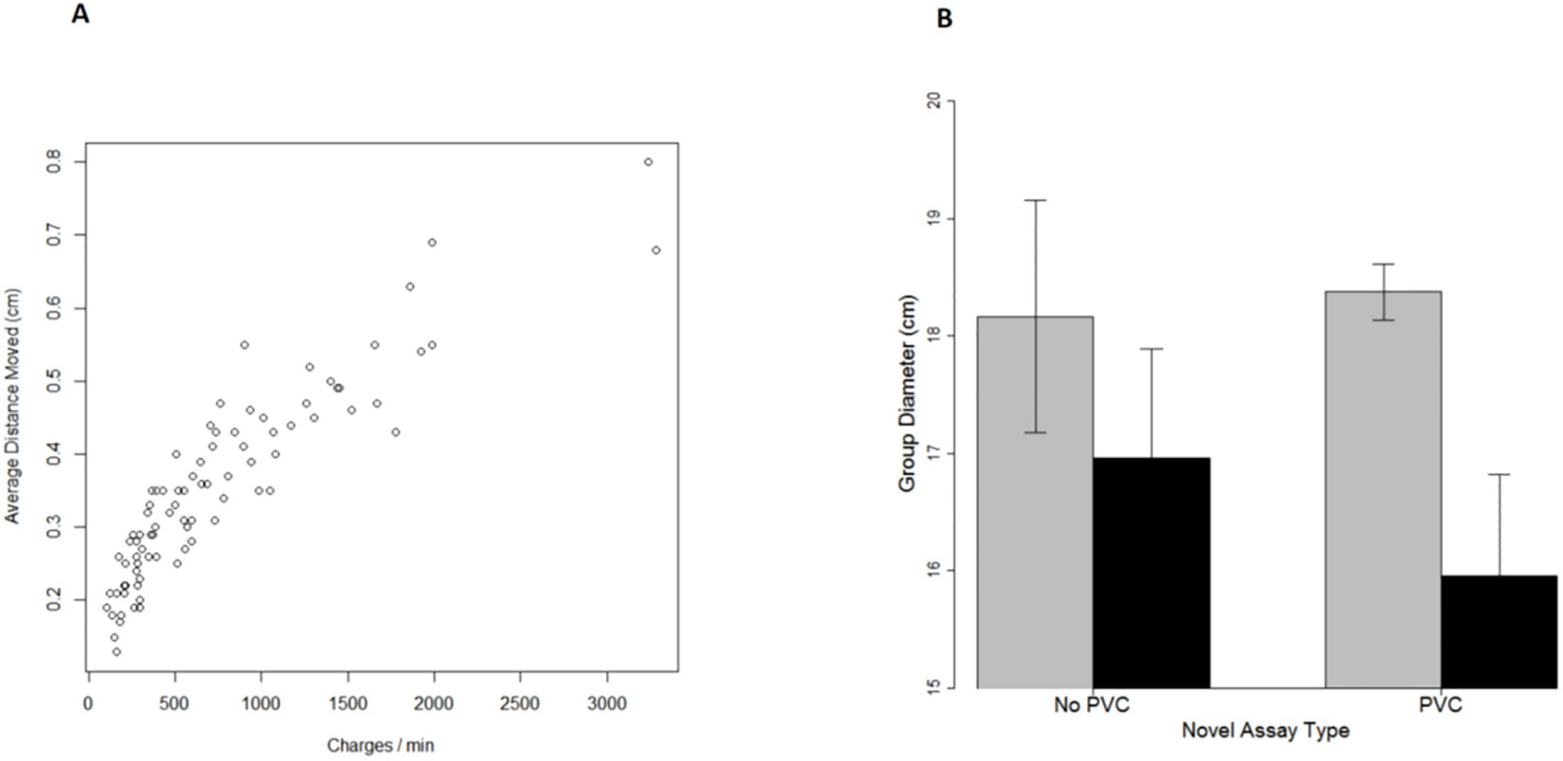
(a) The relationship between *Charge Rate* and *Activity*. (b) Zebrafish groups with recent experience in a more-complex environment (black bars) were not consistently more active than were those with recent experience in a less-complex environment (gray bars). Instead, fish groups tested in a novel PVC context (two bars on the right) were less active than were fish groups tested in a novel empty arena (two bars on the left) (*F*_1,51_ = 6.0, *P* < 0.02). Error bars represent ± 1 standard error.

As expected, *Charge Rate* was tightly linked to *Activity*, as measured by distance moved (Fig 3a, *r* = 0.9, df = 83, *P* << 0.01). Nevertheless, differences in *Activity* explained little, if any, of the effects of recent experience on social behavior. Zebrafish with experience in a more-complex physical environment were not significantly more active than were fish with experience in a less-complex physical environment (*F*_1,28_ = 0.8, *P* = 0.38; Fig 3b). *Activity* was better predicted by assay type. Zebrafish were less active when assayed in a novel arena with cut PVC pipe than when they were assayed in an empty arena, leading to a significant, within-group, effect of assay type (*F*_1,51_ = 6.0, *P* = 0.02). All other main, interaction and within-group effects were not statistically significant (*P* > 0.2).

All three of our behavioral measures were moderately repeatable (*r* = 0.4, df = 28, *P* = 0.02 to 0.04). Zebrafish groups that charged often, were highly active, or had small group diameters in one assay type, behaved similarly in the same assay type two days later.

## Discussion

Our results suggest that social behavior in new contexts can depend on recent experiences, and that the impact of physical environment on adult social behavior can persist even as animals move into new habitats. After housing in more-complex physical environments, zebrafish shoaled more tightly together, suggesting that the primary persistent effect is a shift in spacing patterns. We also found that zebrafish that had been housed for two weeks in a more-complex physical environment charged marginally more often in a novel arena than did those that had been maintained in less-complex contexts. The effect depended to some extent on the immediate context in which social behavior was measured, but was not well explained by differences in activity level.

Our results that shoal cohesion is an important mechanism mediating the impact of physical environment on social behavior also highlight the role of spacing patterns. Animals can reach very high densities with closely-packed spacing patterns, for example, in urban habitats, where the density increases are associated also with shifts in social and anti-predator behavior [2]. High density and the consequently increased competition between conspecifics can have negative impacts on foraging, development and reproduction [52, 53]. Tight spacing can also lead to direct behavioral interference as in bats that forage less efficiently when other bats come too close [54]. Even relatively subtle changes in spacing may lead to detectable shifts in social behavior [26]. Here, we found that the increased interactions between fish that had experienced more complex physical contexts was associated with forming tighter shoals, perhaps the persistent consequence of clustering more closely together in between elements of the complex habitat. Note that shoal diameters in our study fell well within the range of those observed in the wild [38]. It is not clear whether the lack of increased clustering or aggression in our PVC assay is due primarily to the specific type of habitat complexity or to the novelty of that new habitat. Predation risk [55], developmental context [56], evolutionary-genetic background [57], familiarity [58, 59], and chemical properties of the habitat [60] are also important features that can modulate the impact of habitat structure on shoal cohesion. Future work should be aimed at exploring the mechanisms by which previous habitat experience influences social behavior.

Studies have disagreed on the impact of a complex physical environment on aggression. Some have found that increased vegetation is associated with increased aggression [22, 45], perhaps because the vegetation provides simple landmarks that help some individuals to monopolize an area [46, 47]. Others have found decreased aggression in complex, vegetated, habitats [19, 48], perhaps because these studies have measured aggression after allowing dominance-subordinate relationships to stabilize [49, 50] or because of differences in perceived predation risk [51]. Here, we add the finding that the impact of environmental complexity on behavior may depend on the specific type of complexity and the context in which behavior is tested. We found fewer charges in arenas that were made more complex by adding cut PVC-pipe, despite finding increased charges after a two-week experience in a more-complex environment that included plastic vegetation and a refuge. In this case, the novelty of the testing context may be a more important factor than complexity in terms of changing the ways in which fish interact with their environments. In another recent study, we found that the impact of physical complexity also changes over time as animals become more familiar with their physical and social contexts [61]. More detailed analyses of the temporal and social mechanisms are needed to identify exactly what features of the environment facilitate or impede aggression.

Although physical and social environments may have their greatest effects early in development, the brain is plastic throughout the lifetime of the organism [62, 63, 64], and persistent changes in behavior may be the result of shifts in the adult brain. Recent studies have shown that experiences in particular social contexts can impact adult [18, 65] as well as developing [11, 66] brains. For example, male guppies housed with a conspecific female have larger brains than those kept with another male [67], and locusts living in gregarious groups have larger brains than do solitary locusts [68]. Physical context also has an immediate impact on adult behavior, and early experience in particular physical contexts (“enrichment”) can alter brain development [69]. For example, young fish housed in simpler or empty physical contexts have smaller brains [70], decreased brain mRNA expression and spatial learning as adults compared to fish developing in complex environments [56, 71]. Although examples of adult brain growth in fish are rampant, there is also evidence of adult neurogenesis in mammals and in birds [72–74]. Furthermore, some examples in rats and mice are due to the effects of recent experience in more complex, “enriched” physical environments [75, 76]. Our results suggest that similar shifts may occur in adult zebrafish, and that future studies of gene expression in zebrafish brains may yield important insights into contextual, seasonal, and other forms of adult plasticity.

We have shown that there are recent experience effects on social behavior. Our results emphasize the impact of recent experiences on adult social behavior in novel habitats, and highlight the importance of physical environment in creating changes in behavior.

## Supporting information

S1 File Recent experience effects of habitat-full dataset. (XLSX)
